# Nuclei multiplexing with barcoded antibodies for single-nucleus genomics

**DOI:** 10.1038/s41467-019-10756-2

**Published:** 2019-07-02

**Authors:** Jellert T. Gaublomme, Bo Li, Cristin McCabe, Abigail Knecht, Yiming Yang, Eugene Drokhlyansky, Nicholas Van Wittenberghe, Julia Waldman, Danielle Dionne, Lan Nguyen, Philip L. De Jager, Bertrand Yeung, Xinfang Zhao, Naomi Habib, Orit Rozenblatt-Rosen, Aviv Regev

**Affiliations:** 1 Klarman Cell Observatory, Broad Institute of Harvard and MIT, Cambridge, MA 02142 USA; 2 Center for Immunology and Inflammatory Diseases, Division of Rheumatology, Allergy, and Immunology Massachusetts General Hospital and Harvard Medical School, Boston, MA 02129 USA; 3 Center for Translational & Computational Neuroimmunology, Columbia University Medical Center, New York, NY 10019 USA; 4 BioLegend Inc., San Diego, CA 92121 USA; 5 Howard Hughes Medical Institute, Koch Institute of Integrative Cancer Research, Department of Biology, Massachusetts Institute of Technology, Cambridge, MA 02139 USA; 6 Present Address: Department of Biological Sciences, Columbia University, New York, NY 10027 USA; 7 Present Address: Center for Immunology and Inflammatory Diseases, Division of Rheumatology, Allergy, and Immunology, Massachusetts General Hospital and Harvard Medical School, Boston, MA 02129 USA; 8 Edmond and Lily Safra Center for Brain Sciences, Hebrew University of Jerusalem, Jerusalem, 9190401 Israel

**Keywords:** Biological techniques, Biotechnology, Computational biology and bioinformatics, Developmental biology, Genetics

## Abstract

Single-nucleus RNA-seq (snRNA-seq) enables the interrogation of cellular states in complex tissues that are challenging to dissociate or are frozen, and opens the way to human genetics studies, clinical trials, and precise cell atlases of large organs. However, such applications are currently limited by batch effects, processing, and costs. Here, we present an approach for multiplexing snRNA-seq, using sample-barcoded antibodies to uniquely label nuclei from distinct samples. Comparing human brain cortex samples profiled with or without hashing antibodies, we demonstrate that nucleus hashing does not significantly alter recovered profiles. We develop DemuxEM, a computational tool that detects inter-sample multiplets and assigns singlets to their sample of origin, and validate its accuracy using sex-specific gene expression, species-mixing and natural genetic variation. Our approach will facilitate tissue atlases of isogenic model organisms or from multiple biopsies or longitudinal samples of one donor, and large-scale perturbation screens.

## Abstract (web summary)

Single-nucleus RNA-seq enables interrogation of complex tissues but is limited due to batch effects and processing costs. Here the authors use barcoded antibodies against the nuclear pore complex to label nuclei from distinct samples, and develop a computational tool to assign the sample of origin.

## Introduction

Single-nucleus RNA-seq (snRNA-seq) has become an instrumental method for interrogating cell types, states, and function in complex tissues that cannot easily be dissociated^1–3^. This includes tissues rich in cell types, such as neurons, adipocytes and skeletal muscle cells, archived frozen clinical materials, and tissues that must be frozen to register into specific coordinates. Moreover, the ability to handle minute frozen specimens^4^ has made snRNA-seq a compelling option for large-scale studies from tissue atlases^5,6^ to longitudinal clinical trials and human genetics. However, to maximize the success of such studies, there is a crucial need to minimize batch effects, reduce costs, and streamline the preparation of large numbers of samples.

For single-cell analysis, these goals have recently been elegantly achieved by multiplexing samples prior to processing, which are barcoded either through natural genetic variation^7^, chemical labeling^8,9^ or DNA-tagged antibodies (cell hashing)^10^. These methods have improved technical inter-sample variability by early pooling, lowered the cost per sample by overloading cells per microfluidic run—due to an increased ability to detect and discard co-encapsulated cell multiplets sharing the same bead barcode—and reduced the number of parallel processing steps in large studies. However, such approaches have not been reported for nuclei, which may be more challenging to handle due to the different procedures for tagging, and the possibility of more cross-contamination in preparations in the absence of a cell membrane. While one can apply methods leveraging natural genetic variation^7^ for multiplexing nuclei of non-isogenic samples, isogenic samples would require an additional tag.

Here, we follow on these studies by developing a sample multiplexing method for nuclei (nucleus hashing), using DNA-barcoded antibodies targeting the nuclear pore complex. We show that nucleus hashing does not significantly alter the recovered transcriptome profiles, and develop DemuxEM, a computational tool using the Expectation–Maximalization (EM) algorithm to remove multiplets from analysis and assign singlets to their sample of origin. Nucleus-hashing allows us to increase the number of nuclei loaded onto the microfluidic channel, and thus both reduces the cost per nucleus profiled and allows pooling of isogenic samples, such as from isogenic mouse models, multiple specimens from the same human donor, or tissues sampled and preserved from a single individual over time.

## Results

### Nucleus hashing yields faithful expression profiles

**Fig. 1:**
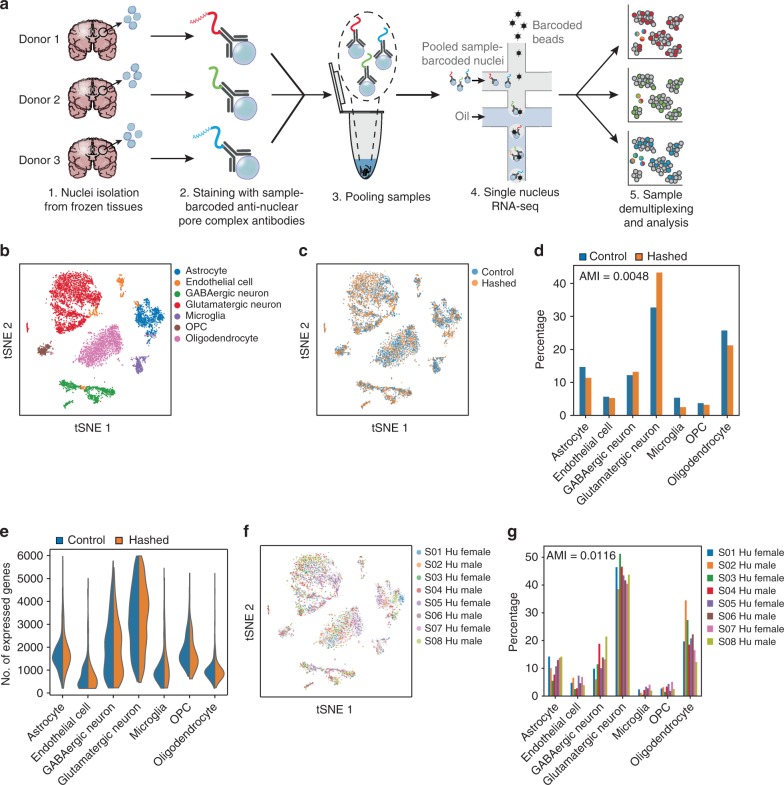
Nuclei multiplexing using DNA-barcoded antibodies targeting the nuclear pore complex. **a** Experimental workflow. Nuclei are isolated from frozen tissues and stained with DNA-barcoded antibodies targeting the nuclear pore complex (MAb414, Biolegend). The DNA barcode encodes a unique sequence representing each tissue sample, enabling sequence-based identification of each nucleus after pooling and profiling the different samples. **b**–**e** Hashed and non-hashed samples of the human cortex from eight postmortem donors yield comparable results. **b** *t*-stochastic neighborhood embedding (tSNE) of single-nucleus profiles (dots) colored by cell type. **c** tSNE as in **b** colored by type of protocol. Non-hashed control sample (blue) and hashed sample (orange) show similar patterns. **d** Cell-type frequencies observed for hashed (orange) and non-hashed control (blue) samples. The adjusted mutual information (AMI) is shown at the top left. **e** Distributions of the number of expressed genes (*y-* axis, left) in each cell type (*x-*axis) in **b**, for nuclei from hashed (orange) and non-hashed control (blue) samples. **f**, **g** Hashed single nuclei from all donors are similarly represented across cell-type clusters. **f** tSNE as in **b** colored by donor. **g** Observed frequencies (*y-*axis) of each cell type (*x-*axis) per donor (color). The adjusted mutual information (AMI) is shown at the top left. Please follow the Supplementary Note in the Supplementary Information to reproduce this figure. Availability of source data is indicated in the Data Availability statement

We isolated nuclei from fresh-frozen murine or human cortex, stained them with antibodies, which target the nuclear pore complex, and which are conjugated to a single-stranded DNA oligo that encodes a sample-specific barcode. We pooled samples prior to droplet encapsulation for single-nucleus RNA-seq (snRNA-seq) (Fig. 1a). The DNA barcodes on the antibodies contain a polyA tail, thus acting as artificial transcripts that register the same bead barcode as nuclear transcripts, coupling the transcription profile to the sample of origin.

The additional antibody labeling and washing steps in our protocol typically introduce some loss of nuclei (average yield ~33%, Supplementary Data 1), but did not alter the quality of transcriptional profiling compared with non-hashed snRNA-seq, in a side-by-side comparison of a hashed (antibody labeled) vs. non-hashed pool of the cortex nuclei derived from eight human donors (Supplementary Data 1). We combined the expression profiles from both hashed and non-hashed data sets, followed by clustering and post hoc annotation with legacy cell-type-specific signatures (Fig. 1b), recovering the cell types previously reported for such samples^1^ (see the Methods section). Both hashed and non-hashed nuclei were similarly represented across the recovered cell types (Fig. 1c), with an adjusted mutual information score of 0.0048 between cell types and experimental conditions (Fig. 1d, Methods), with only slight differences, such as a weak enrichment of glutamatergic neurons in the hashed sample, and similar cell-type-specific numbers of recovered genes (Fig. 1e). Moreover, hashed and non-hashed nuclei were similarly represented across GABAergic neuron subtypes (Supplementary Fig. 1 and Methods). There were very few significantly differentially expressed genes between control and hashed nuclei within a cell type (Supplementary Data 4, Methods; typically 2–3 orders of magnitude less than between cell types, Supplementary Data 4). In most cases, the few genes differentially expressed between hashed and non-hashed nuclei were not enriched for any functional categories (Supplementary Data 5, Methods). For GABAergic neurons, glutamatergic neurons, and oligodendrocytes, mitochondrial genes were significantly downregulated in the hashed nuclei, suggesting that the additional washing steps for the hashing reduced cytosolic debris. For microglia, genes related to synapse organization, nervous system development, cell adhesion, and neurogenesis are significantly upregulated in the hashed nuclei, suggesting this cell type may be more sensitive to manipulation. Each cell type had nuclei from all eight donors (Fig. 1f) with only slightly differing frequencies (Fig. 1g), as expected for a diverse donor cohort^1^ (Supplementary Data 1).

Modifying the staining and washing buffers for nucleus hashing (Methods) compared with those used in cell hashing^10^, improved the transcriptional similarity with the non-hashed control (Supplementary Fig. 2a), and achieved a similar number of genes expressed per nucleus as the non-hashed control (Supplementary Fig. 2b), whereas a PBS-based buffer (used in cell hashing^10^) generally had poorer performance (Supplementary Fig. 2c). We thus performed all experiments with these optimized staining and washing buffers, except those with mouse samples, which were performed prior to buffer optimization. Collectively, these findings indicate that hashing preserves library quality and cell-type distributions.

### DemuxEM computationally demultiplexes hashed nuclei

**Fig. 2:**
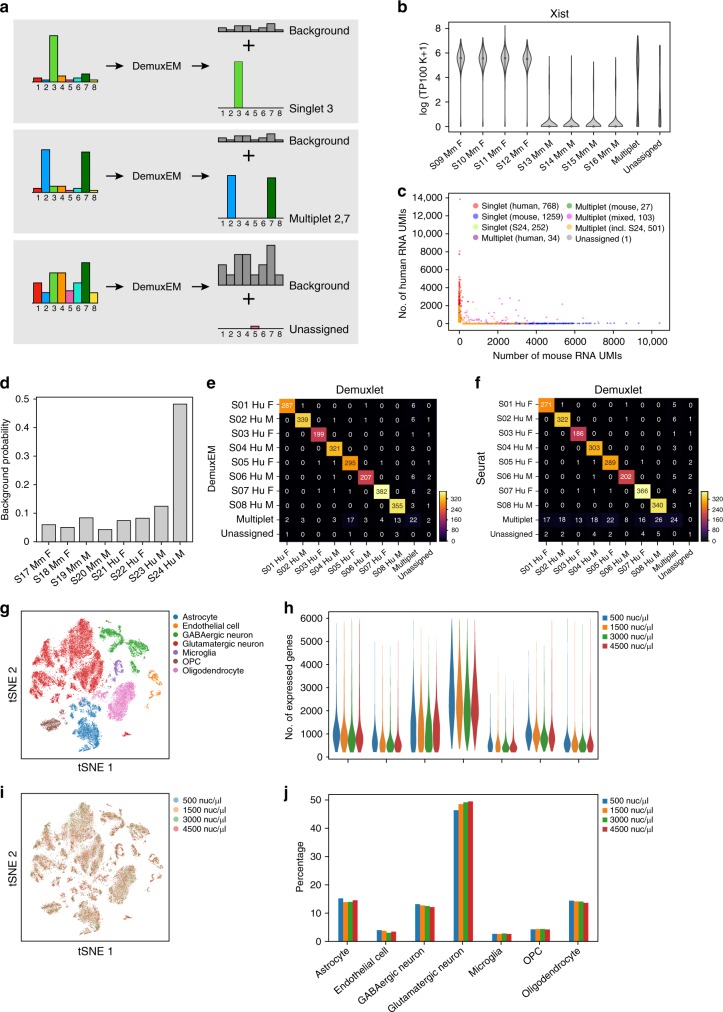
Sample assignment by DemuxEM allows overloading of hashed samples. **a** DemuxEM takes as input for each nucleus a count vector of hashtag UMIs (left) and estimates it as the sum of a background count vector (right, gray histograms) and a signal sample assignment count vector (right, color histograms). Schematic examples: singlet (top), multiplet (middle), and unassigned (bottom). **b** Validation by sex mixing in isogenic mice. Distribution of *Xist* expression (*y-*axis, log(TP100K + 1)) from eight (1–4 females, 5–8 males) cortex samples that were pooled. There is 94.8% agreement between DemuxEM-assigned sample identities of singlets and *Xist* expression. **c**, **d** Species mixing of the human and mouse cortex nuclei. **c** Species-mixing plot. Each nucleus (dot) is plotted by the number of RNA UMIs aligned to pre-mRNA mouse mm10 (*x-*axis) and human GRCh38 (*y-*axis) references (Methods), and colored by its DemuxEM-predicted identity for singlet human (red), singlet mouse (blue), or different multiplets (intra-species: green (mouse) and purple (human); inter-species: fuchsia). S24 singlets (chartreuse) and multiplets (any multiplet that includes a nucleus from sample S24, orange) are colored separately due to its large contribution to ambient hashtags. **d** Distribution of ambient hashtags matching the sample DNA barcode (*x*-axis) identified S24 as a disproportionate contributor. **e**, **f** Validation of hashtag-based assignment by natural genetic variation. Shown is the number of nuclei classified as singlet, multiplet or unassigned (rows, columns) by either natural genetic variation (columns) with Demuxlet^7^, or hashtag UMIs (rows), with (**e**) DemuxEM or (**f**) Seurat^11^. The agreement between Demuxlet and DemuxEM or Seurat is 96 and 92%, respectively. **g**–**j** Nucleus hashing enables overloading. **g** tSNE of combined singlets of eight hashed human cortex samples profiled at loading concentrations of 500, 1500, 3000, or 4500 nuclei/μl. Each nucleus (dot) is colored by its cell type. **h** Comparable distributions of the number of expressed genes (*y-*axis) in each cell type (*x-*axis) in **g**. **i** tSNE as in **g**, colored by loading concentration. **j** Comparable frequencies (*y-*axis) across cell types in **g** (*x-*axis). Please follow the Supplementary Note in the Supplementary Information to reproduce this figure. Availability of source data is indicated in the Data Availability statement

To probabilistically assign each nucleus to its sample barcode, we developed DemuxEM, an EM-based tool (Fig. 2a). For each nucleus, DemuxEM takes as input a vector of hashtag Unique Molecular Identifier (UMI) counts from that nucleus (Fig. 2a, left). A hashtag UMI is a uniquely sequenced combination of the sample-specific DNA barcode from the oligonucleotide conjugated to the antibody, and the cellular barcode and random UMI sequence from the oligonucleotide that was originally bound to the hydrogel bead. The input vector is a mixture of signal hashtag UMIs, which reflect the nucleus’ sample of origin, and background hashtag UMIs, which likely reflect ambient sample barcodes. Hashtag UMIs from the background have different probabilities of matching each of the sample barcodes. DemuxEM estimates this background distribution of sample barcodes based on hashtag UMIs in empty droplets, which are likely to only contain background hashtag UMIs. With this background distribution as a reference, DemuxEM uses an EM algorithm to estimate the fraction of hashtag UMIs from the background in the given droplet and then infer the signal hashtag UMIs by deducting the estimated background UMIs from the input vector. Once the signal has been identified, DemuxEM determines if this droplet encapsulated a single nucleus or a multiplet. For bead barcodes with low-signal hashtag UMIs (e.g., <10 hashtag UMIs), DemuxEM cannot determine the origin of the nucleus and marks it as “unassigned” (Methods).

### Accurate demultiplexing by sex- and individual-mixing

To assess our confidence in calling the sample origin of hashed nuclei by their sample barcodes, we next applied DemuxEM to pooled nuclei of male and female isogenic mice or of human and mouse, such that the single-nucleus transcriptomes provided an orthogonal measure of the sample of origin. First, we multiplexed nuclei isolated from two isogenic C57BL/6J mouse cortices, four technical replicates from each of a female and male mouse (Methods). For DemuxEM-identified singlets, there was a 94.8% agreement between DemuxEM-assigned sample hashtag identities and the expression level of Xist, a transcript predominantly expressed in females (Fig. 2b).

Next, we multiplexed eight cortex samples, four from mouse and four from human (Supplementary Data 1), comparing DemuxEM assignments as human or mouse singlets to their positions in a species-mixing plot based on their number of RNA UMIs mapping to the human or mouse transcriptome (Methods, Fig. 2c). Overall, nuclei assigned by DemuxEM as human or mouse singlets (Fig. 2c, red and blue, respectively) express predominantly human or mouse reads, respectively (Fig. 2c, alignment along the *y*- and *x*-axis). DemuxEM-predicted multiplets occur both on the species-specific axes for intra-species multiplets (Fig. 2c, green (mouse) and purple (human)) and off-axes for inter-species multiplets (Fig. 2c, fuchsia). Notably, DemuxEM’s assignments were robust to changes in the definition of background distribution, Dirichlet prior concentration parameter on the samples, and sequencing depth (Supplementary Fig. 3 and Methods).

We further leveraged the hashtags to address the sources of ambient hashtags in a pool of samples. In general, nuclei dissociated from tissue samples may be at risk of having higher levels of ambient hashtags compared with single-cell hashing, because the cytoplasm is disrupted during lysis and nonspecific antibody binding to the cytosolic content or tissue-derived debris could contribute to the background. Inspection of sample-specific contribution to the hashtag background distribution showed that one of the human samples (S24, Supplementary Data 1) contributed disproportionally to the background (Fig. 2d), suggesting that this sample might have been of lower quality. This donor sample (S24) indeed had the lowest RNA integrity number (RIN) and the highest postmortem interval (PMI) of all subjects in the study (Supplementary Data 1). The ability to identify which samples contribute to the background is an additional benefit of sample hashing, and can help determine quality parameters for sample inclusion.

Next, we validated our hashtag-based demultiplexing with Demuxlet^7^, an approach based on natural genetic variation. We observed excellent agreement between the two methods for the eight human cortex samples (Fig. 2e): 96% of nuclei were predicted with the same identity by both DemuxEM and Demuxlet. Similarly, for Seurat, a package that includes single-cell hashing analysis^11^, 92% of nuclei were predicted with the same identity by both Seurat and Demuxlet (Fig. 2f; Supplementary Data 2).

DemuxEM also offers an improved estimation of the multiplet rate. The multiplet rate per 10x microfluidic channel when loading 7000 cells is expected to be ~3.1%^12^. When pooling eight samples with equal proportions, there are 56 possible *inter*-sample doublet configurations and 8 possible intra-sample ones (the proportion of higher order multiplets is much lower), such that 87.5% (56/64) of the doublets are expected to contain nuclei from multiple samples, which can be identified by our hashing strategy. Since we loaded 7000 nuclei, we expect a detectable multiplet rate of around 2.7% (3.1 * 87.5%). DemuxEM, Demuxlet, and Seurat predicted multiplet rates of 2.8%, 2.1%, and 6.5%, respectively (Supplementary Data 2).

### Hashing-enabled overloading lowers cost per nucleus

DemuxEM’s ability to more accurately detect droplets that encapsulated multiple inter-sample nuclei allowed us to load a higher concentration of nuclei for a given undetectable multiplet rate, thereby significantly lowering the cost per nucleus. To assess how “overloading” a higher concentration of nuclei affects library quality and cell-type distributions, we hashed and pooled another eight human cortex samples (Supplementary Data 1) and loaded a 10x channel with 14 μl of either ~500 nuclei/μl, 1500 nuclei/μl, 3000 nuclei/μl, or 4500 nuclei/μl. When sequencing these libraries at similar depth per nucleus, we recovered similar numbers of expressed genes per nucleus for the different cell types (Fig. 2g, h). Moreover, nuclei from each loading concentration had similar transcriptional states (Fig. 2i) and maintained the same relative cell-type frequencies (Fig. 2j). As expected, the proportion of multiplets increases with increased loading density (Supplementary Fig. 4). Notably, nucleus multiplets do not typically show higher numbers of RNA UMIs compared with singlets (Supplementary Fig. 4), in contrast to cell hashing^10^. The lowest overall cost per nucleus (including nucleus-hashing antibodies, 10x library preparation and sequencing) was achieved for loading 14 μl of 3000 nuclei/μl, resulting in the detection of 13,578 single nuclei in a single 10x channel with an overall ~56% cost per nucleus reduction in our pricing structure, compared with the non-hashed loading density of 500 nuclei/μl (Methods; Supplementary Data 3), albeit with some increase in background signal. Notably, these cost savings can also be achieved by splitting an individual sample into multiple hashed samples, when a larger number of nuclei per sample is required, while still benefitting from the reduced cost and reduced batch effects.

## Discussion

Nucleus hashing is a principled method for multiplexing single nuclei. It reduces batch effects and costs, and helps streamline large experimental studies. DemuxEM is a computational tool we developed that enables accurate multiplet detection, nucleus identity assignment, and identification of the sources of ambient hashtag contamination. As nuclei, rather than cells, become the starting point of many additional methods—especially in epigenomics—it is likely that hashing can be extended to other single-nucleus genomics assays. Together, nucleus hashing and DemuxEM allow us to reliably interrogate cell types, cellular states, and functional processes in complex and archived tissues at a much larger scale than previously possible.

## Methods

### Human samples

The studies were conducted under Rush University IRB approvals L91020181 and L86121802. We have complied with all relevant ethical regulations and informed consent was obtained. We used frozen brain tissue banked by two prospective studies of aging: the Religious Order Study (ROS) and the Memory and Aging Project (MAP), which recruit non-demented older individuals (age > 65)^13^. For the species-mixing experiment, we used four posterior cingulate cortex samples from ROS (Supplementary Data 1). For the human nucleus hashing and overloading experiments, we used 16 dorsolateral prefrontal cortex samples from ROS and MAP (Supplementary Data 1). We selected samples with balanced sex ratio and whole-genome sequencing (WGS) data available^14^ (except for sample S24, for which WGS data are not available).

### Mice

All mouse work was performed in accordance with and received ethical approval from the Institutional Animal Care and Use Committees (IACUC) and relevant guidelines at the Broad Institute and MIT, with protocol 0122–10–16. We have complied with all relevant ethical regulations for animal testing and research. Adult female and male C57BL/6J mice, obtained from the Jackson Laboratory (Bar Harbor, ME), were housed under specific-pathogen-free (SPF) conditions at the Broad Institute, MIT animal facilities.

### Mouse tissue collection

Brains from C57BL/6J mice were obtained and split vertically along the sagittal midline. The cerebral cortices were separated, and excess white matter was removed. Cortices were separated into microcentrifuge tubes and frozen on dry ice. Frozen tissue was stored at −80 °C.

### Nuclei isolation, antibody tagging, and snRNA-seq

A fully detailed, step-by-step protocol is described in Supplementary Methods. Briefly, we thawed and minced tissue, dounced it in lysis buffer, filtered the lysate, and resuspended it in staining buffer. A brief incubation with Fc receptor-blocking solution is followed by incubation with the TotalSeq Hashtag antibodies against the nuclear pore complex (MAb414, catalogue numbers listed in Supplementary Data 1) at 1 μg/100 μl and three washes in ST-SB. Next, nuclei were counted and their concentration normalized to the desired loading concentration and pooled right before running the 10x Genomics single-cell 3’ v2 assay (with minor adjustments listed in Supplementary Methods), followed by library preparation and Illumina sequencing.

### Buffer optimization

In cell-hashing experiments^10^, staining is performed with a PBS-based staining buffer (SB: 2% BSA, 0.02% Tween-20 in PBS). We initially used this buffer during staining for nucleus hashing as well (gender-specific expression and species-mixing experiments)^10^. To further optimize our protocol, we compared both a PBS-based staining buffer and a Tris-based staining buffer (ST-SB, see Supplementary Methods, 2% BSA, 0.02% Tween-20, 10 mM Tris, 146 mM NaCl, 1 mM CaCl_2_, 21 mM MgCl_2_) to a non-hashed control observing better performance in ST-SB, in terms of overall agreement with non-hashed controls and in the number of genes recovered per nucleus (Supplementary Fig. 2). Going forward, we thus performed experiments with these optimized buffers, except for the initial experiments with mouse samples, and recommend to perform the staining and washing steps of nucleus hashing in ST-SB (see Supplementary Methods).

### Nuclei yields

We initially observed significant loss of nuclei during spins and washes, and have mitigated this in part by a combination of the following measures: (1) use of Lo-Bind Eppendorf vials, (2) 2% BSA and 0.02% Tween-20 concentration of the staining and washing buffer, and (3) swing-bucket centrifugation of 1.5 -ml vials to better center the nuclei pellet (compared to centrifugation with fixed angle rotators). Our nuclei counting based on DAPI-staining and transmitted light microscopy before and after hashing shows that we retain on average 33% of the input nuclei (Supplementary Data 1).

### SnRNA-seq data analysis

Starting from BCL files obtained from Illumina sequencing, we ran cellranger mkfastq to extract sequence reads in FASTQ format, followed by cellranger count to generate gene-count matrices from the FASTQ files. Since our data are from single nuclei, we built and aligned reads to genome references with pre-mRNA annotations, which account for both exons and introns. Pre-mRNA annotations improve the number of detected genes significantly compared to a reference with only exon annotations^15^. For human and mouse data, we used the GRCh38 and mm10 genome references, respectively. To compare samples of interest (e.g., different loading concentrations), we pooled their gene-count matrices together, and filtered out low-quality nuclei identified based on any one of the following criteria: (1) a total number of expressed genes <200; (2) a total number of expressed genes > = 6000; or (3) a percentage of RNA UMIs from mitochondrial genes > = 10%. We then normalized and transformed the filtered count matrix to natural log space as follows: (1) selected genes that were expressed in at least 0.05% of all remaining nuclei; (2) normalized the count vector of each nucleus such that the total sum of normalized counts from selected genes is equal to 100,000 (transcripts per 100 K, TP100K); (3) transformed the normalized matrix into the natural log space by replacing each normalized count c with $\log (c + 1)$ (log(TP100K+1)). We performed dimensionality reduction, clustering and visualization on the log-transformed matrix using a standard procedure^16,17^. Specifically, we selected highly variable genes^18^ with a z-score cutoff at 0.5, performed PCA on the standardized sub-matrix consisting of only highly variable genes and selected the top 50 principal components (PCs)^19^, clustered the data based on the 50 selected PCs using the Louvain community detection algorithm^20^ with a resolution at 1.3. We identified cluster-specific gene expression by differential expression analyses between nuclei within the cluster and outside of the cluster^16^ using Welch’s *t* test and Fisher’s exact test; controlled false discovery rates (FDR) at 5% using the Benjamini–Hochberg procedure^21^, and annotated putative cell types based on legacy signatures of human and mouse brain cells. We visualized the reduced dimensionality data using tSNE^22^ with a perplexity at 30. Note that in experiments 1 and 4 (Supplementary Data 1), we identified one cluster that did not express any known cell-type markers and had the lowest median number of RNA UMIs among all clusters. We removed it from further analysis, and repeated the above analysis workflow, except the low-quality nucleus filtration step.

### Sub-cluster analysis of GABAergic neurons

We conducted a sub-cluster analysis on the identified GABAergic neuron nuclei from Fig. 1b. To find the sub-clusters, we selected highly variable genes using the GABAergic neuron subset, performed PCA on the highly variable genes, and found sub-clusters based on top 50 PCs using the Louvain algorithm with a resolution at 1.2.

### Generation of hashtag UMI count matrix

We implemented generate_count_matrix_ADTs, a fast C++ program to extract hashtag UMI count matrix (hashtag by cell barcode) from raw FASTQ files. This program scans each read pair for the valid sequence structure: read1 = 16 nt cell barcode + 10 nt UMI, read2 = 15 nt hashtag + BAAAAAAA + extra bases, and simultaneously records the number of distinct UMIs for each valid cell barcode and hashtag combination based on read pairs with a valid sequence structure. To be robust to sequencing errors, we allowed up to one mismatch for matching cell barcodes, three mismatched for matching hashtags, and one mismatch for matching the poly(A) tail (BAAAAAAA). To speed up the matching process, we encoded each cell barcode, UMI, or hashtag into an 8-byte unsigned integer (2 bits per nucleotide).

### DemuxEM

Suppose we multiplex *n* samples together. For each droplet, we have a count vector of hashtag UMIs from each sample, $(c_1, \cdots ,c_n)$. Each hashtag UMI in the vector can either originate from a properly stained nuclear pore complex (signal) or come from ambient hashtag UMIs (background). We define ${\mathbf{\Theta }} = (\theta _0,\theta _1, \cdots ,\theta _n)$, where $\theta _0$ is the probability that a hashtag UMI is from the background, and $\theta _1, \cdots ,\theta _n$ are the probabilities that the hashtag UMI is true signal $1, \ldots ,n$. If a hashtag UMI is from the background, we denote ${\mathbf{P}} = \left( {p_1, \cdots ,p_n} \right)$ as the probabilities that this hashtag UMI matches the barcode sequence of samples $1, \cdots ,n$. In addition, we require $\mathop {\sum }\limits_{i = 1}^n p_i = 1$.

The probability of generating a hashtag UMI that matches sample *i*’s barcode sequence is:

1 $${\mathrm{P}}\left( {{\mathrm{hashtag}} = i} \right) = \theta _0 \cdot p_i + \theta _i$$

And the log-likelihood of generating the hashtag UMI vector is:

2 $$L\left( {\mathbf{\Theta }} \right) = \mathop {\sum }\limits_{i = 1}^n c_i\log \left( {\theta _0p_i + \theta _i} \right) + \log \frac{{\left( {\mathop {\sum }\nolimits_{i = 1}^n c_i} \right)!}}{{\mathop {\prod }\nolimits_{i = 1}^n c_i!}}$$

DemuxEM estimates two sets of parameters: (1) the background distribution $= \left( {p_1, \cdots ,p_n} \right)$, and (2) ${\mathbf{\Theta }} = (\theta _0,\theta _1, \cdots ,\theta _n)$.

We estimate the background distribution using empty droplets. To identify empty droplets, we first collect all bead barcodes with at least one hashtag UMI. We then calculate the total number of hashtag UMIs each collected bead barcode has and performed a *k*-means clustering with *k* = 2 on the vector of hashtag UMI numbers. The cluster with a lower mean hashtag UMI number was identified as empty droplets. If we denote the set of identified empty droplets as *B*, we can estimate the background distribution as follows:

3 $$p_i = \frac{{\mathop {\sum }\nolimits_{j \in B} c_{ji}}}{{\mathop {\sum }\nolimits_{j \in B} \mathop {\sum }\nolimits_{i = 1}^n c_{ji}}}$$

where $c_{ji}$ is the number of hashtag UMIs matching sample *i* in bead barcode *j*.

We estimate ${\mathbf{\Theta }}$ using an EM algorithm. First, we impose a sparse Dirichlet prior on ${\mathbf{\Theta }}$, ${\mathbf{\Theta }}\sim {\mathrm{Dir}}(1,0, \cdots ,0)$, to encourage the background distribution to explain as much hashtag UMIs as possible. We then follow the EM procedure below:

- E step:
  4 $$z_i = c_i \cdot \frac{{\theta _i}}{{\theta _0p_i + \theta _i}},i = 1, \cdots ,n$$
  5 $$z_0 = \mathop {\sum }\limits_{i = 1}^n c_i \cdot \frac{{\theta _0p_i}}{{\theta _0p_i + \theta _i}}$$
- M step:

6 $$\theta _i = \frac{{\max (z_i - 1,0)}}{{z_0 + \mathop {\sum }\nolimits_{i = 1}^n \max (z_i - 1,0)}},i = 1, \cdots ,n$$

7 $$\theta _0 = \frac{{z_0}}{{z_0 + \mathop {\sum }\nolimits_{i = 1}^n \max (z_i - 1,0)}}$$

Once we have ${\mathbf{\Theta }}$ estimated, we first calculate the expected number of signal hashtag UMIs:

8 $$c_{\mathrm{s}} = \left( {1 - \theta _0} \right) \cdot \mathop {\sum }\limits_{i = 1}^n c_i$$

If $c_{\mathrm{s}} < 10$, the hashtag UMI vector contains too little signal and thus we mark this droplet as “unassigned”. Otherwise, we count the number of samples that has at least 10% signal hashtag UMIs, $|\left\{ {i{\mathrm{|}}\frac{{\theta _i}}{{1 - \theta _0}} \ge 0.1} \right\}|$. If this number is 1, the droplet is a singlet. Otherwise, it is a multiplet.

### DemuxEM robustness analysis

We assessed the robustness of DemuxEM using the 2509 hashed nuclei of Fig. 1b. By default, DemuxEM uses *k*-means clustering with a random state of 0 to identify the background distribution and sets the Dirichlet prior concentration parameters of the *n* samples to 0 (i.e., ${\mathrm{\alpha }}_1 = \ldots = {\mathrm{\alpha }}_{\mathrm{n}} = 0$). We perturbed parameters related to the definition of the background distribution, the choice of prior, and the sequencing depth, and calculated the consistency between the perturbed and the default DemuxEM results, where consistency is defined as the percentage of nuclei that are predicted to have the same singlet/multiplet type and sample identities by DemuxEM with both default and perturbed settings. (1) Different definition of the background distribution. (a) *k*-means’ random_state parameter sets the seed of the random number generator that is used for centroid initialization. Different random_state corresponds to different random centroid initialization. We ran DemuxEM with ten randomly generated random_state parameters. (b) We used a different clustering algorithm, hierarchical agglomerative clustering (HAC), to identify the background distribution. Since the standard HAC algorithm has a time complexity of ${\mathrm{O}}(n^3)$ and we have over 552,363 cell barcodes with nonzero hashtag UMI counts, it is computationally infeasible to apply HAC to all cell barcodes. Thus, we grouped hashtag UMI counts into 500 bins. The first 499 bins contain 1106 counts each, and the last bin contains the largest 469 counts. We calculated the mean of each bin to produce a small data set of only 500 data points. We then applied the HAC algorithm on this data set to get two clusters. We tried HAC with four different linkage criteria: ward, complete linkage, average linkage, and single linkage. Once we got the cluster label for each bin, we assigned cell barcodes within that bin the same cluster label. (c) We tested a simple thresholding strategy to identify the background distribution. Given a threshold *x*, the background consists of all cell barcodes with hashtag UMI counts $\le x$. We tried ten different thresholds: 10, 20, 30, 40, 50, 60, 70, 80, 90, and 100. (2) Choice of Dirichlet prior concentration parameter on samples. We tested DemuxEM with ten different Dirichlet concentration parameters for the samples—0.1, 0.2, 0.3, 0.4, 0.5, 0.6, 0.7, 0.8, 0.9, and 1.0. (3) Sequencing depth. We sub-sampled the hashtag UMI count matrix using Bernoulli sampling with 9 different *p* parameters: 0.9, 0.8, 0.7, 0.6, 0.5, 0.4, 0.3, 0.2, and 0.1, and ran DemuxEM on each subsampled count matrix.

### Cell-type-specific differential gene expression analysis

We conducted differential expression analysis between 2509 hashed nuclei and 3574 control nuclei of Fig. 1b for 27,947 genes within each of the seven cell-type clusters: astrocyte, endothelial cells, GABAergic neurons, glutamatergic neurons, microglia, oligodendrocyte precursor cells (OPC), and oligodendrocytes. We used the Mann–Whitney U test to calculate the *P*-value for each gene and controlled the false discovery rate (FDR) at 0.05 using the Benjamini–Hochberg procedure^21^. The differentially expressed (DE) genes for each cell type are listed in Supplementary Data 4. In addition, we summarized the total number of DE genes for each cell type in Supplementary Data 4. We applied the same differential expression detection procedure to the 3574 control nuclei to calculate the number of DE genes between control nuclei of one cell type and all other control nuclei for each cell type, which is shown in Supplementary Data 4 as well.

### Gene ontology enrichment analysis

We applied Fisher’s exact test in PANTHER^11^ with an FDR correction (FDR = 0.05) on the up and down DE genes (separately) in Supplementary Data 4 for each cell type. The analysis results are available in Supplementary Data 5.

### Preprocessing of the species-mixing data

The species-mixing cDNA library is deeply sequenced. According to the Cell Ranger report, this library has a sequencing saturation of 89.8%, such that on average ten reads correspond to 1 UMI [https://kb.10xgenomics.com/hc/en-us/articles/115003646912-How-is-sequencing-saturation-calculated-]. In contrast, other libraries in this study have read-to-UMI ratios of 3 or 4 (Supplementary Data 1). Deeply sequenced libraries tend to produce more PCR chimeras, which lead to reads with cell barcodes and mRNA sequences from different nuclei^23^. Since PCR chimeras are more likely to be produced at later stages of PCR, they tend to have a smaller read-to-UMI ratio than normal cDNA molecules^23^. Thus, in order to remove PCR chimeras, we only kept UMIs with at least two reads in this data set.

### Demuxlet-based demultiplexing

Demuxlet requires a list of SNPs as inputs. We called germline variants for each human donor from WGS data by following GATK Best Practices recommendations^24,25^, with GATK^26^ v3.4-0-g7e26428. We then selected 2,385,459 biallelic SNPs that have PASS in the FILTER field, VQSLOD scores of at least 22, and non-identical genotypes in 8 human donors as the Demuxlet input. We ran Demuxlet with the following parameters:–field GT–group-list barcodes.txt. barcodes.txt is a file containing cell barcodes that we want Demuxlet to demultiplex. We used the following rules to interpret Demuxlet outputs, which can potentially reduce the number of false positive doublets (Chun (Jimmie) Ye, personal communication). We first looked at the BEST column of Demuxlet-produced.best file. For any cell barcode, if its BEST column starts with (1) SNG-, it is a singlet; (2) DBL- and the PRB.DBL column $\le 0.99$, it is a singlet; (3) DBL- and the PRB.DBL column $> 0.99$, it is a doublet; (4) AMB, it is an unassigned droplet.

### Seurat-based demultiplexing

We first extracted the hashtag-count matrix from sequenced hashtag reads using CITE-seq-count v1.3 [https://hoohm.github.io/CITE-seq-Count/] with the following parameters: -t antibody_index.csv -wl 737K-august-2016.txt -cbf 1 -cbl 16 -umif 17 -umil 26 -tr “^[ATGC]{15}[TGC][A]{6,}”. In the parameter list, antibody_index.csv maps sample-specific hashtag barcodes to sample names and 737K-august-2016.txt is the cell barcode whitelist for 10x Genomics single-cell 3’ v2 chemistry. We then ran Seurat’s HTODemux with default parameters on the hashtag-count matrix to demultiplex interested cell barcodes.

### Estimation of cost per single nucleus when overloading

We estimate the reduction in cost per single nucleus for a given pricing structure, assuming *X* for loading one 10x channel, *Y* for sequencing one HiSeq lane, and *Z* for the TotalSeq nuclei hashtag cost per hashed sample, to allow readers to determine the costs for their own pricing structures. We sequenced four HiSeq lanes in total for four overloading experiments, with proportions roughly as 1:3:6:9 (500 nuc/µl:1500 nuc/µl:3000 nuc/µl:4500 nuc/µl). Based on these values, the sequencing costs for the four settings are $\frac{4}{{19}}Y$, $\frac{{12}}{{19}}Y$, $\frac{{24}}{{19}}Y$, and $\frac{{36}}{{19}}Y$, respectively. Adding the 10x channel cost of *X*, and the TotalSeq nuclei hashtag costs of 8*Z*, the final cost for each setting is $X + \frac{4}{{19}}Y + 8Z$, $X + \frac{{12}}{{19}}Y + 8Z$, $X + \frac{{24}}{{19}}Y + 8Z$, and $X + \frac{{36}}{{19}}Y + 8Z$, respectively. We then divide each cost by the total number of singlets we detected (Supplementary Data 3) to obtain cost per single nucleus in each overloading setting.

## Supplementary information

Supplementary Information

Supplementary Data 1

Supplementary Data 2

Supplementary Data 3

Supplementary Data 4

Supplementary Data 5

Description of Additional Supplementary Files

Reporting Summary

## Data Availability

Raw mouse sequencing data are available from the Sequence Read Archive with accession numbers SRR8703773 (RNA reads) and SRR8703774 (hashtag reads). Processed and raw mouse expression data are available from the Single Cell Portal [https://portals.broadinstitute.org/single_cell/study/SCP377/experiment-2-mouse-pbs]. This is the source data underlying Fig. 2b. Raw human sequencing data are shared widely with a data use agreement through the RADC Resource Sharing Hub [www.radc.rush.edu]. Whole-Genome Sequencing data for the human samples can be obtained through the National Institute of Aging supported AMP-AD Knowledge Portal [https://www.synapse.org/#!Synapse:syn2580853/wiki/409840] by searching keyword ROSMAP. Processed human expression data are available from the Single Cell Portal [https://portals.broadinstitute.org/single_cell/study/SCP375/experiment-1-stonly, https://portals.broadinstitute.org/single_cell/study/SCP379/experiment-3-human-mouse-pbs-clust, https://portals.broadinstitute.org/single_cell/study/SCP381/experiment-4-human-st, https://portals.broadinstitute.org/single_cell/study/SCP371/experiment-1-all]. This is the source data underlying Figs. 1, 2c-j, and Supplementary Figs. 1–4.

## Code availabilty

DemuxEM is released as part of the scCloud for single cell analysis. Its source codes are available at: https://github.com/broadinstitute/scRNA-Seq/tree/master/scCloud/scCloud/demuxEM. We also prepared a docker image that reproduces figures and tables in this paper, which is available at: https://hub.docker.com/r/regevlab/demuxem. Please follow the Supplementary Note in the Supplementary Information to run the docker.

## References

[1] Habib N (2017). Massively parallel single-nucleus RNA-seq with DroNc-seq. Nat. Methods.

[2] Nagy, C. et al. Single-nucleus RNA sequencing shows convergent evidence from different cell types for altered synaptic plasticity in major depressive disorder. *BioRxiv*, 10.1101/384479 (2018).

[3] Lake BB (2018). Integrative single-cell analysis of transcriptional and epigenetic states in the human adult brain. Nat. Biotechnol..

[4] Habib N (2016). Div-Seq: single-nucleus RNA-Seq reveals dynamics of rare adult newborn neurons. Science.

[5] Sunkin SM (2013). Allen Brain Atlas: an integrated spatio-temporal portal for exploring the central nervous system. Nucleic Acids Res.

[6] Smillie, C. S. et al. Rewiring of the cellular and inter-cellular landscape of the human colon during ulcerative colitis. *BioRxiv*, 10.1101/455451 (2018).

[7] Kang HM (2018). Multiplexed droplet single-cell RNA-sequencing using natural genetic variation. Nat. Biotechnol..

[8] Gehring, J., Park, J. H., Chen, S., Thomson, M. & Pachter, L. Highly multiplexed single-cell RNA-seq for defining cell population and transcriptional spaces. *BioRxiv*, 10.1101/315333 (2018).

[9] McGinnis, C. S. et al. MULTI-seq: scalable sample multiplexing for single-cell RNA sequencing using lipid-tagged indices. *BioRxiv*, 10.1101/387241 (2018). 10.1038/s41592-019-0433-8 PMC6837808 31209384

[10] Stoeckius M (2018). Cell Hashing with barcoded antibodies enables multiplexing and doublet detection for single cell genomics. Genome Biol..

[11] Butler A, Hoffman P, Smibert P, Papalexi E, Satija R (2018). Integrating single-cell transcriptomic data across different conditions, technologies, and species. Nat. Biotechnol..

[12] 10x Genomics. What is the maximum number of cells that can be profiled? https://kb.10xgenomics.com/hc/en-us/articles/360001378811-What-is-the-maximum-number-of-cells-that-can-be-profiled-

[13] Bennett DA (2018). Religious orders study and rush memory and aging project. J. Alzheimers Dis..

[14] De Jager PL (2018). A multi-omic atlas of the human frontal cortex for aging and Alzheimer’s disease research. Sci. Data.

[15] Bakken TE (2018). Single-nucleus and single-cell transcriptomes compared in matched cortical cell types. PLoS One.

[16] Wolf FA, Angerer P, Theis FJ (2018). SCANPY: large-scale single-cell gene expression data analysis. Genome Biol..

[17] Shekhar K (2016). Comprehensive classification of retinal bipolar neurons by single-cell transcriptomics. Cell.

[18] Macosko EZ (2015). Highly parallel genome-wide expression profiling of individual cells using nanoliter droplets. Cell.

[19] Zheng GX (2017). Massively parallel digital transcriptional profiling of single cells. Nat. Commun..

[20] Traag VA (2015). Faster unfolding of communities: speeding up the Louvain algorithm. Phys. Rev. E Stat. Nonlin Soft Matter Phys..

[21] Benjamini Y, Hochberg Y (1995). Controlling the false discovery rate—a practical and powerful approach to multiple testing. J. R. Stat. Soc. B Met.

[22] van der Maaten, L. & Hinton, G. Visualizing data using t-SNE. *J. Mach. Learn. Res. ***9**, 2579–2605 (2008).

[23] Dixit, A. Correcting chimeric crosstalk in single cell RNA-seq experiments. *BioRxiv*, 10.1101/093237 (2016).

[24] DePristo MA (2011). A framework for variation discovery and genotyping using next-generation DNA sequencing data. Nat. Genet..

[25] Van der Auwera GA (2013). From FastQ data to high confidence variant calls: the Genome Analysis Toolkit best practices pipeline. Curr. Protoc. Bioinforma..

[26] McKenna A (2010). The Genome Analysis Toolkit: a MapReduce framework for analyzing next-generation DNA sequencing data. Genome Res..

