# Author Correction: Nuclei multiplexing with barcoded antibodies for single-nucleus genomics

**DOI:** 10.1038/s41467-020-19357-w

**Published:** 2020-11-02

**Authors:** Jellert T. Gaublomme, Bo Li, Cristin McCabe, Abigail Knecht, Yiming Yang, Eugene Drokhlyansky, Nicholas Van Wittenberghe, Julia Waldman, Danielle Dionne, Lan Nguyen, Philip L. De Jager, Bertrand Yeung, Xinfang Zhao, Naomi Habib, Orit Rozenblatt-Rosen, Aviv Regev

**Affiliations:** 1 Klarman Cell Observatory, Broad Institute of Harvard and MIT, Cambridge, MA 02142 USA; 2 Center for Immunology and Inflammatory Diseases, Division of Rheumatology, Allergy, and Immunology Massachusetts General Hospital and Harvard Medical School, Boston, MA 02129 USA; 3 Center for Translational & Computational Neuroimmunology, Columbia University Medical Center, New York, NY 10019 USA; 4 BioLegend Inc., San Diego, CA 92121 USA; 5 Howard Hughes Medical Institute, Koch Institute of Integrative Cancer Research, Department of Biology, Massachusetts Institute of Technology, Cambridge, MA 02139 USA; 6 Present Address: Department of Biological Sciences, Columbia University, New York, NY 10027 USA; 7 Present Address: Center for Immunology and Inflammatory Diseases, Division of Rheumatology, Allergy, and Immunology, Massachusetts General Hospital and Harvard Medical School, Boston, MA 02129 USA; 8 Present Address: Edmond and Lily Safra Center for Brain Sciences, Hebrew University of Jerusalem, Jerusalem, 9190401 Israel

**Keywords:** Biological techniques, Biotechnology, Computational biology and bioinformatics, Developmental biology, Genetics

Correction to: *Nature Communications* 10.1038/s41467-019-10756-2, published online 02 July 2019.

In the original version of this article, the raw human sequencing data was available at the RADC Resource Sharing Hub through a data use agreement. This data can now be accessed at the Synapse AMP-AD Knowledge Portal [https://www.synapse.org/#!Synapse:syn22213200]. This has now been corrected in the PDF and HTML versions of the paper. Additionally, Yiming Yang was incorrectly associated with ‘Department of Biological Sciences, Columbia University, New York, NY, 10027, USA’ and Naomi Habib was incorrectly associated with ‘Center for Immunology and Inflammatory Diseases, Division of Rheumatology, Allergy, and Immunology, Massachusetts General Hospital and Harvard Medical School, Boston, MA, 02129, USA’ in the HTML version of the paper. The PDF was correct at the time of publication. This has now been corrected in the HTML version of the paper.

