# Supplementary material for: Nuclei multiplexing with barcoded antibodies for single-nucleus genomics
Source: Nat Commun. 2019 Jul 2;10:2907. doi: 10.1038/s41467-019-10756-2 (PMC6606589; doi:10.1038/s41467-019-10756-2)
Supplement: Supplementary file 1 — Supplementary Information [file 41467_2019_10756_MOESM1_ESM.pdf]

Nuclei multiplexing with barcoded antibodies for single-nucleus genomics

Gaublomme J.T., Li B. et al.

SUPPLEMENTARY INFORMATION

Supplementary Figures 1-4

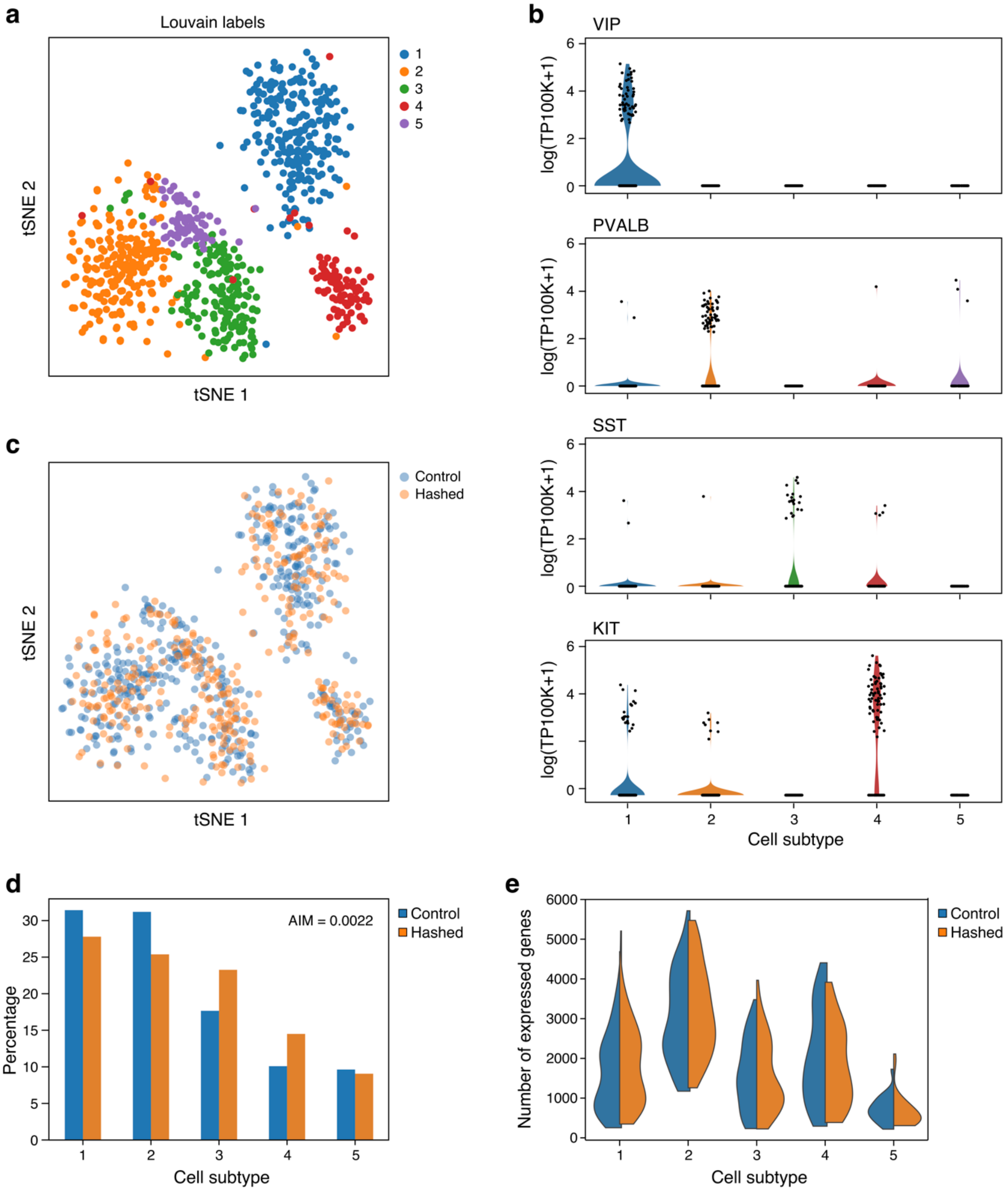

Supplementary Figure 1 | Hashed and non-hashed samples are similarly represented across GABAergic neuron subtypes. Sub-clustering analysis was performed on the GABAergic neuron nuclei from Figure 1b (Methods). **a** tSNE of single nucleus profiles colored by sub-cluster labels. **b** Expression distributions of 4 GABAergic neuron subtype markers[1] across 5 sub-clusters. Sub-clusters 1-4 expressed subtype-specific markers VIP, PVALB, SST and KIT respectively. **c-e** Hashed and non-hashed nuclei are similarly represented across GABAergic neuron subtype clusters. **c** tSNE as in a colored by type of protocol. **d** Sub-cluster frequencies observed for hashed (orange) and non-hashed control (blue) samples. The adjusted mutual information (AMI) is shown in the top right. **e** Distributions of the number of expressed genes (*y* axis, left) in each sub-cluster (*x* axis) in a, for nuclei from hashed (orange) and non-hashed control (blue) samples. Please follow the Supplementary Note in the Supplementary Information to reproduce this figure. Availability of source data is indicated in the Data Availability statement

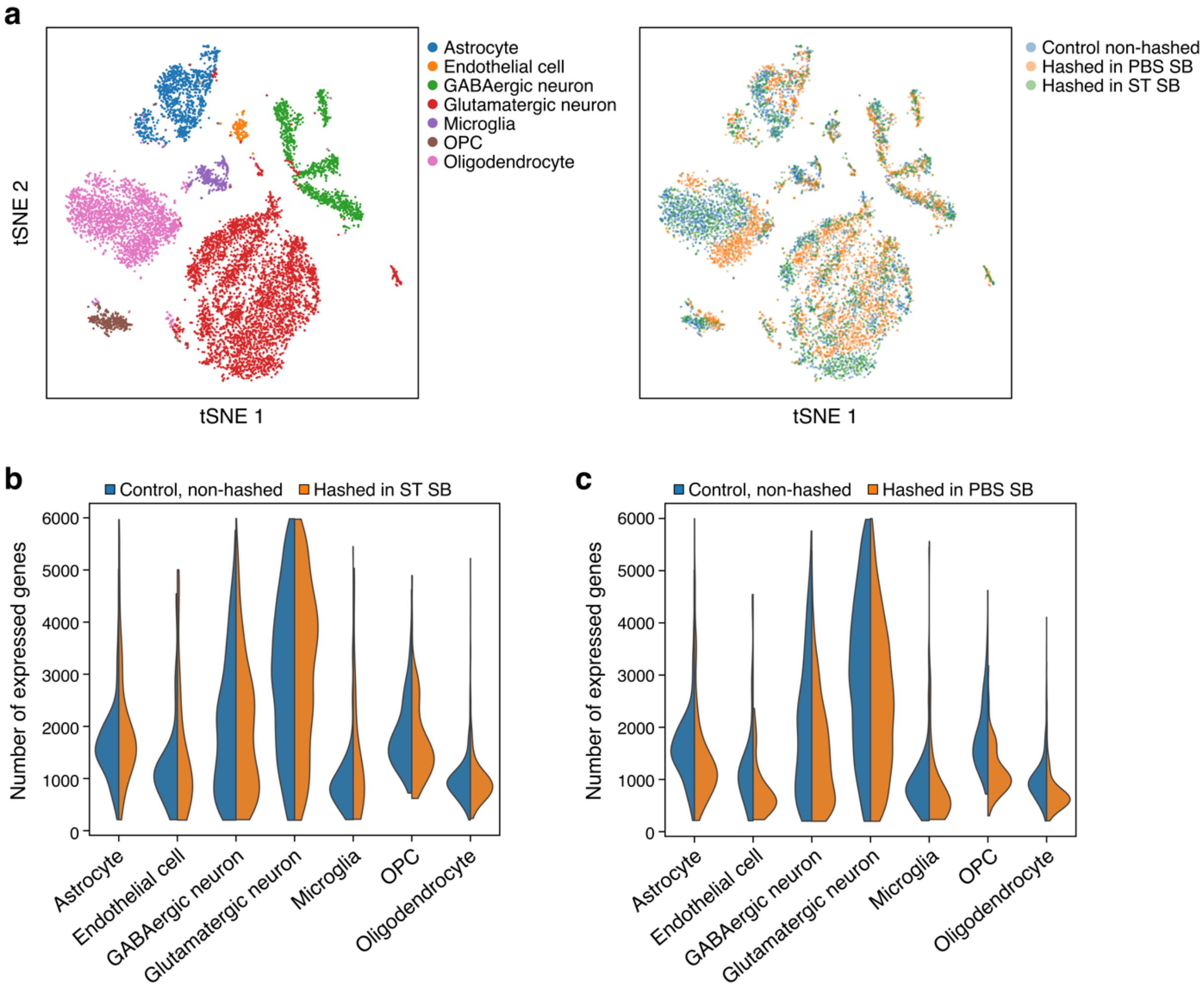

Supplementary Figure 2 | Buffer optimization for multiplexing. a tSNE of single nucleus profiles from non-hashed control, PBS-based (PBS-SB) and ST-based staining buffer (ST-SB) colored by either cell type (left) or protocol (right). Nuclei stained with ST-SB buffer (green) largely overlap with the non-hashing control nuclei (blue), whereas PBS-stained nuclei (orange) show some separation within the clusters. **b-c** decreased number of expressed genes detected when using PBS-SB. Distribution of number of expressed genes (*y* axis) across cell types (*x* axis) for nuclei stained with ST-SB (**b,** orange) or PBS-SB (**c**, orange) compared to the non-hashing control (blue). Except for microglia, ST-SB performs better across cell types. Please follow the

Supplementary Note in the Supplementary Information to reproduce this figure. Availability of source data is indicated in the Data Availability statement

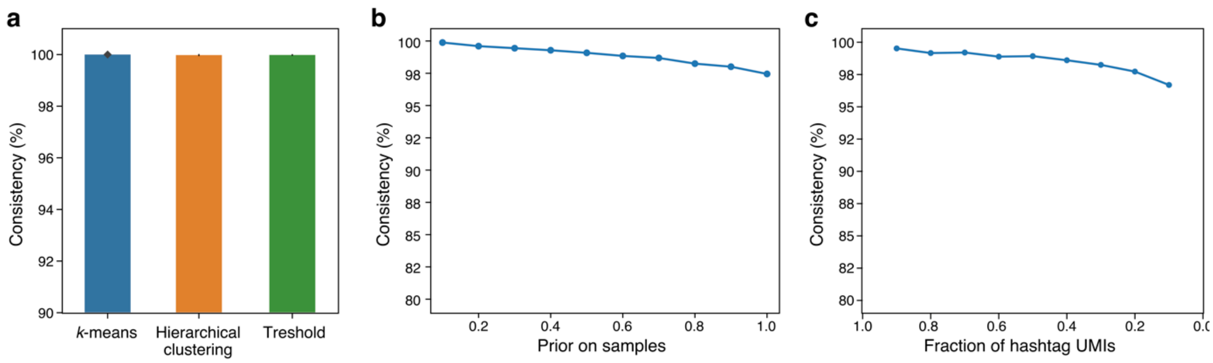

Supplementary Figure 3 | DemuxEM robustness. Shown is the consistency of the assignments (percentage nuclei predicted to have the same singlet/multiplet type and sample identities, *y* axis) produced by DemuxEM on the hashed nuclei in **Figure 1b** with default settings *vs*. different perturbed settings. **a** Different background distribution estimates: *k*-means algorithm with different random state, hierarchical agglomerative clustering algorithm with different linkages, and different hard thresholds. Error bars represent 95% confidence intervals. **b** Increased Dirichlet prior concentration parameters on the samples (*x* axis). **c** Decreased sequencing depths (*x* axis). Please follow the Supplementary Note in the Supplementary Information to reproduce this figure. Availability of source data is indicated in the Data Availability statement

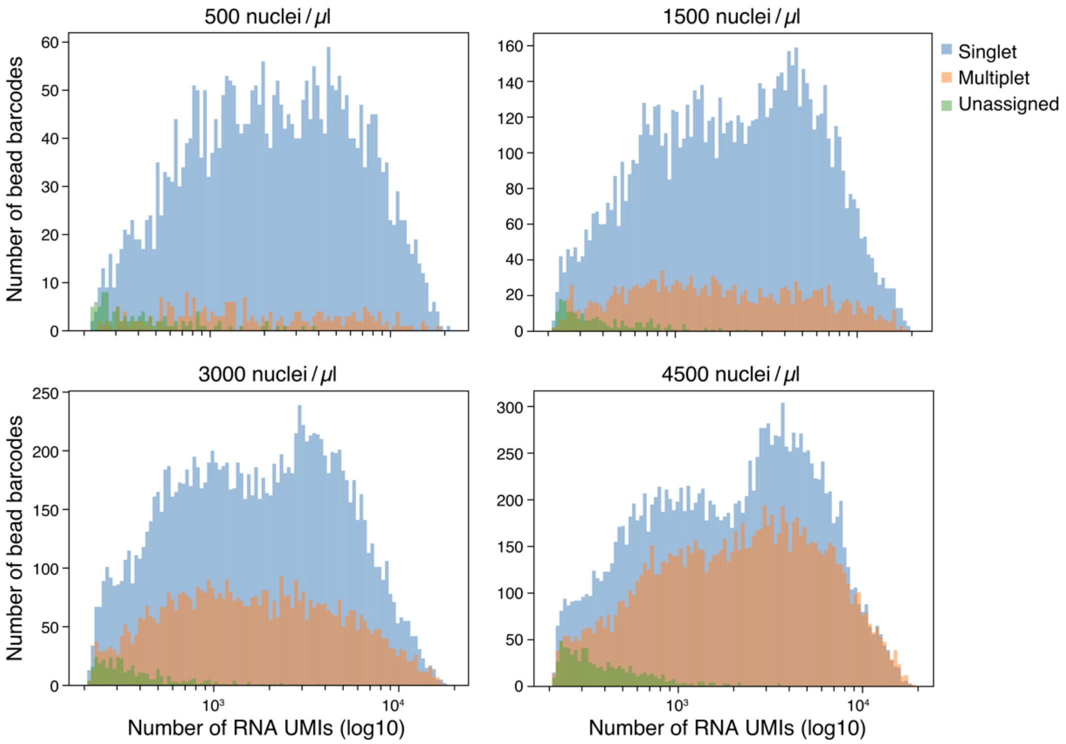

Supplementary Figure 4 | Percentage of multiplets increases with increased loading concentrations. Distribution of number of bead barcodes (*y* axis) for beads with different numbers of detected UMIs (*x* axis, log 10 scale), for singlets (blue), multiplets (orange) and unassigned droplets (green), in 8 hashed human cortex samples loaded at concentrations of 500, 1,500, 3,000 or 4,500 nuclei/µl. Although the multiplet rate rises with increasing loading concentrations, we observe similar RNA UMI count distributions between singlets and multiplets, a feature not observed for single-cell hashing[2]. Please follow the Supplementary Note in the Supplementary Information to reproduce this figure. Availability of source data is indicated in the Data Availability statement

Supplementary Methods

Detailed Protocol for nucleus hashing.

Materials

NAME	CATALOG #	VENDOR
BSA-Molecular Biology Grade - 12 mg	B9000S	New England Biolabs
Dounce homogenizers	D8938-1SET	Sigma
Pre-Separation Filters (30 µm)	130-041-407	Miltenyi Biotec
Pre-Separation Filters (20 µm)	130-101-812	Miltenyi Biotec
Eppendorf® LoBind microcentrifuge tubes	Z666505-100EA	Sigma Aldrich
Human TruStain FcX™	422302	BioLegend
Beckman Coulter SPRI SELECT REAGENT 5ML	NC0406406	Fisher Scientific
KAPA HiFi HotStart ReadyMix	NC0465187	Fisher Scientific

1. **Prepare buffers fresh**

NP40 Lysis Buffer (NST): 0.1% NP40, 10mM Tris, 146mM NaCl, 1mM $CaCl_2$, 21mM $MgCl_2$, 40U/mL of RNAse inhibitor

ST Wash Buffer: (10mM Tris, 146mM NaCl, 1mM $CaCl_2$, 21mM $MgCl_2$), 0.01% BSA (NEB B9000S), 40U/mL of RNAse inhibitor

ST Staining buffer (ST-SB): 2%BSA, 0.02%Tween-20, 10mM Tris, 146mM NaCl, 1mM $CaCl_2$, 21mM $MgCl_2$)

2. **Tissue lysis and homogenizing**

Nuclei were extracted as previously described[2] with the following minor modifications:

a) For each sample to barcode and pool: prepare a separate homogenizer and douncing pestles A & B. Add 1ml NST buffer to the dounce homogenizer and keep on ice.

Note: Keep tissues/homogenate and buffers on ice throughout the protocol. Pre-cool the centrifuge to 4C and keep at 4C for all steps.

b) Cut a 50-200mg section of frozen brain tissue with a scalpel and dissect to remove white matter and vasculature. Mince tissue and add it to the homogenizer.

c) with a total volume of 1mL, dounce 20 times with pestle A followed by 20 times with pestle B.

d) Add 1ml of ST buffer, filter through 30µm filters (Milentyi Biotec 130-041-407) and transfer filtered homogenate to a 15mL tube.

e) Rinse the homogenizer with 3x 1ml of ST buffer, filter through 30µm filters and add to the filtered homogenate to add up to a final volume of 5ml.

f) Immediately spin down at 500g for 5 mins at 4C to pellet the nuclei in swing bucket rotor

g) Remove supernatant

h) Resuspend nuclei in 200µl of ST-SB, filter with 20um (miltenyibiotec 130-101-812) and transfer to a lo-bind 1.5ml tube (Sigma-Aldrich, Z666505-100EA)

Count nuclei

Nuclei were counted using the Nexcelom Cellometer Vision 10x objective and a DAPI stain.

a) DAPI was diluted to 2.5µg/µl in ST Buffer.
b) 20µl of the DAPI was pipet mixed with 20ul of the nuclei suspension and 20µl was loaded onto a cellometer cell counting chamber of standard thickness (Nexcelom catalog number: CHT4-SD100-002) and counted using a custom assay with the dilution factor set to 2.

Hashtag antibody staining

Note: this part mirrors the cell-hashing protocol [1], with very minor differences.

a) Add 10 µl Fc Blocking reagent (Biolegend 422302) per 1-2M of nuclei in 100µl of ST-SB/nuclei and incubate for 5 minutes at 4C.

b) Add 1 µg of single nuclei hashing antibody (MAb414) per 100µl of ST-SB/nuclei mix and incubate for 10 minutes at 4C.

c) Wash nuclei 3 times with 1.2 mL ST-SB, spin in swinging bucket rotor for 5 minutes at 500g and 4˚C.

d) Resuspend nuclei in ST-SB at 500-3,000 cells/µl.

e) Filter nuclei through MACS Pre-Separation Filters (20µm), and count nuclei to verify concentration after filtration. Adjust to desired concentration.

f) Pool all samples at desired proportions and immediately proceed to next step.

10X Genomics single-nuclei sequencing

Use 14µl of pooled sample as input into the 10X Genomics single-cell 3' v2 assay and process as described until before cDNA amplification.

Library preparation

a) To increase yield of HTO products during the 10X Genomics cDNA amplification step: Add 1µl of 2µM HTO PCR additive primer (5'GTGACTGGAGTTCAGACGTGTGC*T*C)

b) After cDNA amplification: Separate HTO-derived cDNAs (<180bp) and mRNA-derived cDNAs (>300bp). Perform SPRI selection to separate mRNA-derived and antibody-oligo-derived cDNAs. DO NOT DISCARD SUPERNATANT FROM 0.6X SPRI. THIS CONTAINS THE HASHTAGS.

c) Add 0.6X SPRI (Beckman Coulter, B23317) to cDNA reaction as described in 10X Genomics protocol.

d) Incubate 5 minutes and place on magnet. Supernatant contains hashtags, and beads contain full length mRNA-derived cDNAs.

Library preparation for mRNA-derived cDNA >300bp (bead fraction)

Proceed with standard 10X protocol for cDNA sequencing library preparation.

Library preparation for mRNA-derived cDNA <300bp (supernatant fraction)

Purify Hashtags using two 2X SPRI purifications per manufacturer protocol:

● Add 1.4X SPRI to supernatant to obtain a final SPRI volume of 2X SPRI.

● Transfer entire volume into a low-bind 1.5mL tube.

● Incubate 10 minutes at room temperature.

● Place tube on magnet and wait ~2 minutes until solution is clear.

● Carefully remove and discard the supernatant.

● Add 400 µl 80% ethanol to the tube without disturbing the pellet and stand for 30 seconds (only one ethanol wash).

● Carefully remove and discard the ethanol wash.

● Centrifuge tube briefly and return it to magnet.

● Remove and discard any remaining ethanol.

● Resuspend beads in 50 µl water.

● Perform another round of 2X SPRI purification by adding 100 µl SPRI reagent directly onto resuspended beads.

● Mix by pipetting, and incubate 10 minutes at room temperature.

● Place tube on magnet and wait ~2 minutes until solution is clear.

● Carefully remove and discard the supernatant.

● Add 200 µl 80% ethanol to the tube without disturbing the pellet and let stand for 30 seconds (first Ethanol wash).

● Carefully remove and discard the ethanol wash.

● Add 200 µl 80% ethanol to the tube without disturbing the pellet and let stand for 30 seconds (second Ethanol wash).

● Carefully remove and discard the ethanol wash.

● Centrifuge tube briefly and return it to magnet.

● Remove and discard any remaining ethanol and allow the beads to air dry for 2 minutes (do not over-dry beads).

● Resuspend beads in 90 µl water.

● Mix vigorously by pipetting and incubate at room temperature for 5 minutes.

● Place tube on magnet and transfer clear supernatant into PCR well.

● Prepare 100µL PCR reaction with purified small fraction:

o 45 µl purified Hashtag fraction

o 50 µl 2x KAPA Hifi PCR Master Mix.

o 2.5 µl TruSeq DNA D7xx_s primer (containing i7 index) 10 µM. (i.e. D701: 5'CAAGCAGAAGACGGCATACGAGATCGAGTAAT GTGACTGGAGTTCAGACGTGT*G*C)

o 2.5 µl SI PCR oligo at 10 µM (SI PCR: 5'AATGATACGGCGACCACCGAGATCTACACTCTTTCCCTACACGACGC* T*C)

Cycling conditions:

95˚C 3 min

95˚C 20 sec |

64˚C 30 sec | ~ 8 cycles

72˚C 20 sec |

72˚C 5 min

Perform 1.6X SPRI purification by adding 160 µl SPRI reagent.

● Incubate 5 minutes at room temperature.

● Place tube on magnet and wait 1 minute until solution is clear.

● Carefully remove and discard the supernatant.

● Add 200 µl 80% ethanol to the tube without disturbing the pellet and let stand for 30 seconds (first ethanol wash).

● Carefully remove and discard the ethanol wash.

● Add 200 µl 80% ethanol to the tube without disturbing the pellet and let stand for 30 seconds (second ethanol wash).

● Carefully remove and discard the ethanol wash.

● Centrifuge tube briefly and return it to magnet.

● Remove and discard any remaining ethanol and allow the beads to air dry for 2 minutes.

● Resuspend beads in 20 µl water.

● Pipette mix vigorously and incubate at room temperature for 5 minutes.

● Place tube on magnet and transfer clear supernatant to PCR tube.

Quantify library

Quantify library by standard methods (QuBit, BioAnalyzer). Hashtag library will be around 180 bp.

Sequence

Combine mRNA library and HTO library (~90% mRNA to 10% HTO), and sequence with the regular 10X RNA-seq read structure:

o Read 1 = 26

o Read 2 = 55 bp

o Index 1 = 8 bp

o Index 2 = n/a

Supplementary Note

Instruction to generate Figures from the paper "Nuclei multiplexing with barcoded antibodies for single-nucleus genomics"

Launch regevlab/demuxem docker

1. Install docker by following the instructions [here](https://docs.docker.com/install/)

2. Adjust docker settings to allow at least 8 GiB of Memory and 4 GiB of Swap. Note that by default this docker runs on 8 CPU threads.

3. Pull **regevlab/demuxem** docker to your local computer:

```
docker pull regevlab/demuxem
```

4. Launch docker:

```
docker run -it regevlab/demuxem
```

Description of input files

We generated gene-count matrix from 10x genomics V2 scRNA-Seq data via Google Cloud. In addition, we generated hashtag-count matrix from FASTQ files via Google Cloud.

The table below listed files we downloaded from Google buckets:

File name	Description
/software/inputs/experiment1_human_st_raw_10x.h5	Experiment 1, human, ST-SB buffer, cell ranger produced raw gene-count matrix in hdf5 format
/software/inputs/experiment1_human_st_raw_GRCh38_premrna	Experiment 1, human, ST-SB buffer, folder, cell ranger produced raw gene-count matrix in market format
/software/inputs/experiment1_human_st_ADT.csv	Experiment 1, human, ST-SB buffer, generate_count_matrix_ADTs produced hashtag-count matrix in CSV format
/software/inputs/experiment1_human_control_raw_10x.h5	Experiment 1, human, control, cell ranger produced raw gene-count matrix in hdf5 format
/software/inputs/experiment1_human_pbs_raw_10x.h5	Experiment 1, human, PBS-SB buffer, cell ranger produced raw gene-count matrix in hdf5 format
/software/inputs/experiment1_human_pbs_ADT.csv	Experiment 1, human, PBS-SB buffer, generate_count_matrix_ADTs produced hashtag-count matrix in CSV format
/software/inputs/experiment1_count_matrix.csv	Experiment1, sample sheet for scCloud
/software/inputs/experiment2_mouse_pbs_raw_10x.h5	Experiment 2, mouse, PBS-SB buffer, cell ranger produced raw gene-count matrix in hdf5 format

/software/inputs/experiment2_mouse_pbs_ADT.csv | Experiment 2, mouse, PBS-SB buffer, generate_count_matrix_ADTs produced hashtag-count matrix in CSV format
/software/inputs/experiment3_human_mouse_pbs_raw_10x.h5 | Experiment 3, human & mouse mixture, PBS-SB buffer, cell ranger produced raw gene-count matrix in hdf5 format
/software/inputs/experiment3_human_mouse_pbs_molecule_info.h5 | Experiment 3, human & mouse mixture, PBS-SB buffer, cell ranger produced molecule information file
/software/inputs/experiment3_human_mouse_pbs_ADT.csv | Experiment 3, human & mouse mixture, PBS-SB buffer, generate_count_matrix_ADTs produced hashtag-count matrix in CSV format
/software/inputs/experiment4_human_st_500_raw_10x.h5 | Experiment 4, human, ST-SB buffer, 500 nuc / microliter, cell ranger produced raw gene-count matrix in hdf5 format
/software/inputs/experiment4_human_st_500_ADT.csv | Experiment 4, human, ST-SB buffer, 500 nuc / microliter, generate_count_matrix_ADTs produced hashtag-count matrix in CSV format
/software/inputs/experiment4_human_st_1500_raw_10x.h5 | Experiment 4, human, ST-SB buffer, 1500 nuc / microliter, cell ranger produced raw gene-count matrix in hdf5 format
/software/inputs/experiment4_human_st_1500_ADT.csv | Experiment 4, human, ST-SB buffer, 1500 nuc / microliter, generate_count_matrix_ADTs produced hashtag-count matrix in CSV format
/software/inputs/experiment4_human_st_3000_raw_10x.h5 | Experiment 4, human, ST-SB buffer, 3000 nuc / microliter, cell ranger produced raw gene-count matrix in hdf5 format
/software/inputs/experiment4_human_st_3000_ADT.csv | Experiment 4, human, ST-SB buffer, 3000 nuc / microliter, generate_count_matrix_ADTs produced hashtag-count matrix in CSV format
/software/inputs/experiment4_human_st_4500_raw_10x.h5 | Experiment 4, human, ST-SB buffer, 4500 nuc / microliter, cell ranger produced raw gene-count matrix in hdf5 format
/software/inputs/experiment4_human_st_4500_ADT.csv | Experiment 4, human, ST-SB buffer, 4500 nuc / microliter, generate_count_matrix_ADTs produced hashtag-count matrix in CSV format
/software/inputs/experiment4_count_matrix.csv | Experiment 4, sample sheet for scCloud

Pre-generated intermediate result files

To generate the following intermediate result files, we need to process sequencing reads from FASTQ files. Since we are dealing with human data and we are not allowed to share sequencing data publicly, we only provided the result files and the commands to generate them here.

Intermediate result file	Description
/software/inputs/experiment1_demuxlet.best	Demuxlet result file
/software/inputs/experiment1_human_st_ADT.seurat.csv	Hashtag-count matrix for running Seurat HTODemux

* Run Demuxlet

Suppose we have Demuxlet installed and the path to its executable added to PATH. Also suppose we have the following files in the working directory.

File name	Description
possorted_genome_bam.bam	Aligned BAM file produced by Cell Ranger for experiment 1, human, ST-SB buffer
snps_for_demuxlet.VQSLOD_22.vcf.gz	GZipped genetic variant file prepared according to "Running Demuxlet" section of the Materials and Methods
experiment1_stonly_barcodes_for_demuxlet.txt	A list of cell barcodes used in Figure 1b, this file can be generated by running the scripts in the docker

Command:

```
demuxlet --sam possorted_genome_bam.bam --vcf snps_for_demuxlet.VQSLOD_22.vcf.gz --field GT --group-list experiment1_stonly_barcodes_for_demuxlet.txt --out experiment1_demuxlet
```

* RUN CITE-seq-Count

Suppose we have CITE-seq-Count v3.0 installed and the following files in the working director.

File name	Description
experiment1_human_st_ADT_R1.fastq.gz	Read 1 for hashtags, experiment 1, human, ST-SB buffer
experiment1_human_st_ADT_R2.fastq.gz	Read 2 for hashtags, experiment 1, human, ST-SB buffer
antibody_index.csv	Sample sheet maps
737K-august-2016.txt	10x Genomics V2 chemistry cell barcode white list, can be found from Cell Ranger software package

`antibody_index.csv` content:

```
TTCCTGCCATTACTA,S1HuF
CCGTACCTCATTGTT,S2HuM
GGTAGATGTCCTCAG,S3HuF
TGGTGTCATTCTTGA,S4HuM
ATGATGAACAGCCAG,S5HuF
CTCGAACGCTTATCG,S6HuM
TGACGCCGTTGTTGT,S7HuF
GCCTAGTATGATCCA,S8HuM
```

Command:

```
CITE-seq-Count -R1 experiment1_human_st_ADT_R1.fastq.gz -R2 experiment1_human_st_ADT_R2.fastq.gz -t antibody_index.csv -wl 737K-august-2016.txt -cbf 1 -cbl 16 -umif 17 -umil 26 -tr "^[ATGC]{15}[TGC][A]{6,}" -o experiment1_human_st_ADT.seurat.csv
```

Reproduce figures and tables

Note that due to the randomness of the tSNE algorithm, the tSNE plots generated might be different from the paper. But they should convey the same information.

Table reproduction

File name	Description
table_s1.txt	Reproduction of Columns A, B, G-K of snRNA-Seq stats tab of Table_S1.xlsx
table_s2.txt	Reproduction of Table_S2.xlsx
table_s3.txt	Reproduction of the nuclei numbers in Table_S3.xlsx
table_s4.txt	Reproduction of the DE_gene_summary tab in Table_S4.xlsx
table_s4.de.xlsx	Reproduction of all other tabs in Table_S4.xlsx

Results in Table_S5.xlsx were generated using the PANTHER online server. The input data for the PANTHER are up and down DE genes in Table_S4.xlsx. Please refer to Methods, Gene Ontology enrichment analysis for more details.

Run the following commands to reproduce all figures and appropriate portions of tables

```
python reproduce_figures.py
python reproduce_tables.py
```

When the execution is done, all the output files are in `/software/outputs` folder. Please refer to PDF files starting with "figure_" to check generated figures. Please refer to text files starting with "table_" to check generated tables.

Supplementary References

1 Habib, N. *et al.* Massively parallel single-nucleus RNA-seq with DroNc-seq. *Nat Methods* **14**, 955-958, doi:10.1038/nmeth.4407 (2017).

2 Stoeckius, M. *et al.* Cell Hashing with barcoded antibodies enables multiplexing and doublet detection for single cell genomics. *Genome Biol* **19**, 224, doi:10.1186/s13059-018-1603-1 (2018).
