# Supplementary material for: Nuclei multiplexing with barcoded antibodies for single-nucleus genomics
Source: Nat Commun. 2019 Jul 2;10:2907. doi: 10.1038/s41467-019-10756-2 (PMC6606589; doi:10.1038/s41467-019-10756-2)
Supplement: Supplementary file 7 — Description of Additional Supplementary Files [file 41467_2019_10756_MOESM7_ESM.docx]

**Title: Supplementary Data 1
Description:** Sample information, snRNA-seq statistics and nuclei yield data.

**Title: Supplementary Data 2
Description:** Comparison of droplet assignment between DemuxEM, Demuxlet and Seurat.

**Title: Supplementary Data 3
Description:** Cost per nucleus calculation as a function of nuclei loading concentration.

**Title: Supplementary Data 4
Description:** Cell-type specific differential gene expression analysis.

**Title: Supplementary Data 5
Description:** Gene Ontology enrichment analysis.
