# Supplementary material for: Nuclei multiplexing with barcoded antibodies for single-nucleus genomics
Source: Nat Commun. 2019 Jul 2;10:2907. doi: 10.1038/s41467-019-10756-2 (PMC6606589; doi:10.1038/s41467-019-10756-2)
Supplement: Supplementary file 8 — Reporting Summary [file 41467_2019_10756_MOESM8_ESM.pdf]

nature research

Corresponding author(s): Aviv Regev (aregev@broadinstitute.org)

Last updated by author(s): May 22, 2019

Reporting Summary

Nature Research wishes to improve the reproducibility of the work that we publish. This form provides structure for consistency and transparency in reporting. For further information on Nature Research policies, see Authors & Referees and the Editorial Policy Checklist.

Statistics

For all statistical analyses, confirm that the following items are present in the figure legend, table legend, main text, or Methods section.

n/a	Confirmed	
☐	☒	The exact sample size (*n*) for each experimental group/condition, given as a discrete number and unit of measurement
☐	☒	A statement on whether measurements were taken from distinct samples or whether the same sample was measured repeatedly
☐	☒	The statistical test(s) used AND whether they are one- or two-sided<br>*Only common tests should be described solely by name; describe more complex techniques in the Methods section.*
☐	☒	A description of all covariates tested
☐	☒	A description of any assumptions or corrections, such as tests of normality and adjustment for multiple comparisons
☐	☒	A full description of the statistical parameters including central tendency (e.g. means) or other basic estimates (e.g. regression coefficient) AND variation (e.g. standard deviation) or associated estimates of uncertainty (e.g. confidence intervals)
☐	☒	For null hypothesis testing, the test statistic (e.g. *F*, *t*, *r*) with confidence intervals, effect sizes, degrees of freedom and *P* value noted<br>*Give P values as exact values whenever suitable.*
☐	☒	For Bayesian analysis, information on the choice of priors and Markov chain Monte Carlo settings
☒	☐	For hierarchical and complex designs, identification of the appropriate level for tests and full reporting of outcomes
☐	☒	Estimates of effect sizes (e.g. Cohen's *d*, Pearson's *r*), indicating how they were calculated

Our web collection on statistics for biologists contains articles on many of the points above.

Software and code

Policy information about availability of computer code

Data collection	No software was used.
Data analysis	1) Illumina's bcl2fastq (v2.20) is used for extracting FASTQ files from Illumina's BCL files.<br>2) Cell Ranger (v2.2.0) is used to extract FASTQ files by calling bcl2fastq and generate gene-count matrices.<br>3) scCloud (v0.12.0), developed by Bo Li and his colleagues, is used to perform all single-nucleus RNA-Seq data analysis.<br>4) DemuxEM, which is part of the scCloud package, is used to demultiplex nucleus-hashing data.<br>5) Seurat (v2.3) and Demuxlet are used for benchmarking purposes.

For manuscripts utilizing custom algorithms or software that are central to the research but not yet described in published literature, software must be made available to editors/reviewers. We strongly encourage code deposition in a community repository (e.g. GitHub). See the Nature Research guidelines for submitting code & software for further information.

Data

Policy information about availability of data

All manuscripts must include a data availability statement. This statement should provide the following information, where applicable:

- Accession codes, unique identifiers, or web links for publicly available datasets
- A list of figures that have associated raw data
- A description of any restrictions on data availability

Raw mouse sequencing data are available from the Sequence Read Archive with accession numbers SRR8703773 (RNA reads) and SRR8703774 (hashtag reads).

Processed and raw mouse expression data are available from the Single Cell Portal: https://portals.broadinstitute.org/single_cell/study/SCP377/experiment-2-mouse-pbs.

Raw human sequencing data are shared widely with a data use agreement through the RADC Resource Sharing Hub: www.radc.rush.edu.

Whole Genome Sequencing data for the human samples can be obtained through the AMP-AD Knowledge Portal that is supported by the National Institute of Aging: https://www.synapse.org/ - !Synapse:syn2580853/wiki/409840.

Processed human expression data are available from the Single Cell Portal: https://portals.broadinstitute.org/single_cell/study/SCP375/experiment-1-stonly, https://portals.broadinstitute.org/single_cell/study/SCP379/experiment-3-human-mouse-pbs-clust, https://portals.broadinstitute.org/single_cell/study/SCP381/experiment-4-human-st, https://portals.broadinstitute.org/single_cell/study/SCP371/experiment-1-all.

Field-specific reporting

Please select the one below that is the best fit for your research. If you are not sure, read the appropriate sections before making your selection.

☒ Life sciences ☐ Behavioural & social sciences ☐ Ecological, evolutionary & environmental sciences

For a reference copy of the document with all sections, see nature.com/documents/nr-reporting-summary-flat.pdf

Life sciences study design

All studies must disclose on these points even when the disclosure is negative.

Sample size	For the overloading experiment, we chose overloading size of 7K, 21K, 42K, and 63K nuclei based on past experience.
Data exclusions	Low quality single nucleus were filtered out according to per-established criteria, which are clearly documented in the Methods section.
Replication	For each experiment, we have 8 technical/biological replicates with balanced gender. We clearly documented our replication settings in our manuscript.
Randomization	We do not have experimental groups.
Blinding	We do not have experimental groups.

Reporting for specific materials, systems and methods

We require information from authors about some types of materials, experimental systems and methods used in many studies. Here, indicate whether each material, system or method listed is relevant to your study. If you are not sure if a list item applies to your research, read the appropriate section before selecting a response.

Materials & experimental systems

n/a	Involved in the study	
☐	☒	Antibodies
☒	☐	Eukaryotic cell lines
☒	☐	Palaeontology
☐	☒	Animals and other organisms
☐	☒	Human research participants
☒	☐	Clinical data

Methods

n/a	Involved in the study	
☒	☐	ChIP-seq
☒	☐	Flow cytometry
☒	☐	MRI-based neuroimaging

Antibodies

Antibodies used	monoclonal antibody against the nuclear pore complex, clone Mab414, BioLegend, San Diego, CA
Validation	Single-stranded DNA oligonucleotides containing a 5' amine modification, an Illumina PCR handle (GTGACTGGAGTTCAGACGTGTGCTCTTCCGATCT), unique antibody barcode sequences (15-bp), a poly-A tail, and a phosphorothioate backbone between the last three bases at the 3' end, were purchased (IDT, Coralville, Iowa). Oligonucleotides were directly conjugated to a highly specific, flow cytometry-tested monoclonal antibody against the nuclear pore complex (clone Mab414, BioLegend, San Diego, CA), yielding conjugates that were a mixture of one or two oligos per antibody molecule. Unbound antibody and oligo were removed by SEC. The purity of the antibody oligo conjugates were verified by agarose gel electrophoresis, diluted to a final concentration of 0.5 mg/mL, and stored at 4˚C supplemented with 0.9% sodium azide and 1 mM EDTA.<br><br>Antibody-oligo conjugates were validated by Sanger Sequencing (Eton Biosciences, San Diego, CA) to ensure that the correct oligo was conjugated to the antibody.

Animals and other organisms

Policy information about studies involving animals; ARRIVE guidelines recommended for reporting animal research

Laboratory animals	All mouse work was performed in accordance with the Institutional Animal Care and Use Committees (IACUC) and relevant guidelines at the Broad Institute and MIT, with protocol 0122-10-16. Adult female or male C57BL/6J mice, aged 10-12 weeks, were obtained from the Jackson Laboratory (Bar Harbor, ME), and housed under specific-pathogen-free (SPF) conditions at the Broad Institute, MIT animal facilities.
Wild animals	NA
Field-collected samples	NA
Ethics oversight	NA

Note that full information on the approval of the study protocol must also be provided in the manuscript.

Human research participants

Policy information about studies involving human research participants

Population characteristics	We used post-mortem frozen brain tissue from 10 males and 10 females, in the age range of 76-96 years old, from two prospective studies of aging: the Religious Order Study (ROS) and the Memory and Aging Project (MAP)
Recruitment	Non-demented older individuals (age >65) were recruited to the aforementioned studies.
Ethics oversight	The study was conducted under Rush University IRB approvals L91020181 and L86121802.

Note that full information on the approval of the study protocol must also be provided in the manuscript.
